# Oncological Patients With Endocrine Complications After Immunotherapy With Checkpoint Inhibitors Present Longer Progression-Free and Overall Survival

**DOI:** 10.3389/fonc.2022.847917

**Published:** 2022-03-24

**Authors:** Stavroula A. Paschou, Michael Liontos, Evangelos Eleftherakis-Papaiakovou, Katerina Stefanaki, Christos Markellos, Konstantinos Koutsoukos, Flora Zagouri, Theodora Psaltopoulou, Meletios-Athanasios Dimopoulos

**Affiliations:** ^1^ Endocrine Unit and Diabetes Center, Department of Clinical Therapeutics, Alexandra Hospital, School of Medicine, National and Kapodistrian University of Athens, Athens, Greece; ^2^ Hematology and Oncology Unit, Department of Clinical Therapeutics, Alexandra Hospital, School of Medicine, National and Kapodistrian University of Athens, Athens, Greece

**Keywords:** immunotherapy, endocrine, thyroiditis, diabetes, hypophysitis, cancer

## Abstract

**Aim:**

The aim of this study was to investigate the association of endocrine complications after ICI immunotherapy with progression-free survival (PFS) and overall survival (OS) in a large single-center oncological cohort.

**Patients and Methods:**

In total, 351 patients were included in the analysis, 248 men (70.7%) and 103 women (29.3%). The median age was 66 years. Patients had a variety of cancer types, namely, bladder cancer (131, 37.3%), renal cancer (89, 25.4%), lung cancer (74, 21.1%), ovarian cancer (22, 6.3%), and other types of cancer (35, 10%). The majority (314, 89.4%) were classified as stage IV, while 10.6% (37) were classified as stage III. Most of the patients received immunotherapy with anti-PD1 agents (262, 74.6%) and the rest with anti-PD-L1 agents (89, 25.4%). Kaplan–Meier estimates were used to describe and visualize the effect of categorical variables on OS and PFS. Survival analysis was performed by Kaplan–Meier curves, and survival differences between groups were estimated using the log-rank test. The estimation of the prognostic value of several variables with patients’ survival was made by Cox regression models.

**Results:**

In total, 68 (19.4%) of patients presented an endocrine complication after immunotherapy with ICIs. Specifically, 66 (18.8%) had thyroid dysfunction, 1 patient presented hypophysitis (0.3%), and 1 patient had a combination of thyroid dysfunction and hypophysitis (0.3%). Patients with an endocrine complication had mPFS of 15 months (95% CI 11.0–18.9 months), while in those without endocrine complication mPFS was 7 months (95% CI 6.1–7.9 months, *p* < 0.001). Similarly, median OS (mOS) was statistically significant lower in the patients’ group without endocrine complication. In fact, mOS was 51 months (95% CI 39.3–62.7 months) for these patients. The presence of endocrine complications after immunotherapy with ICIs retained its significance in terms of longer PFS (HR 0.57, 95% CI 0.39–0.81) and OS (HR 0.53, 95% CI 0.32–0.90) after multivariate analysis.

**Conclusions:**

ICI endocrinopathies may be a positive predictor of immunotherapy response.

## Introduction

Immune checkpoints (ICs) are molecules that modulate the duration and amplitude of physiological immune function, playing important roles in maintaining immune homeostasis (1). Immune checkpoint inhibitors (ICIs) are antibodies that target certain immune checkpoints (ICs), such as programmed death 1 (PD-1), its ligand (PD-L1), or cytotoxic T-lymphocyte antigen-4 (CTLA-4) (1, 2). ICIs have emerged as a powerful new tool for various types of cancers, namely, lymphoma, melanoma, lung cancer, renal cell carcinoma, urothelial carcinoma, etc. The indications of ICIs are currently increasing (1–5).

As these ICs are crucial for immunological self-tolerance, such therapies can trigger autoimmune adverse effects, affecting a number of organs (2, 6). Endocrinopathies are among the most common complications, including thyroid dysfunction, hypophysitis, and more rarely diabetes mellitus (DM) and primary adrenal insufficiency (6). The time of onset of endocrinopathies presents a great range (weeks to months after the initial dose) (7, 8). Furthermore, the severity of endocrinopathies presents a great range and grading systems are used in the clinical practice (1, 9). Combination of ICIs appears to increase the risk of endocrine complications (2, 3).

It has been hypothesized that autoimmune complications or immune-related adverse events (irAEs) after ICIs may be a positive predictor of immunotherapy response (10). Particularly, several studies and a recent meta-analysis provided evidence that progression-free survival (PFS) and overall survival (OS) are longer in patients with development of autoimmune disorders in general and of endocrinopathies specifically (11–13). A recent study provided evidenced that the development of autoimmune complications was associated with improved survival in patients with advanced (stage III/IV) NSCLC treated with anti-PD-L1 agents (12). Such autoimmune complications, including endocrine ones, may represent bystander effects from activated T lymphocytes and despite undesirable symptoms can correlate with favorable disease outcomes. This phenomenon is known in the literature as “beneficial autoimmunity” (10, 14).

However, evidence so far is scarce and the exact mechanisms remain elusive. Moreover, not all studies have provided similar results and the topic remains quite controversial (13). As this hypothesis needs further investigation, we aimed to investigate the association of endocrine complications after ICI immunotherapy with PFS and OS in a large single-center oncological cohort.

## Patients and Methods

### Patients and Protocol

All patients who received immunotherapy with ICIs in the Unit of Oncology, Department of Clinical Therapeutics, Alexandra Hospital, School of Medicine, National and Kapodistrian University of Athens in Athens, Greece, from June 2016 to December 2020 were studied. In total, 351 patients were included in the analysis, 248 men (70.7%) and 103 women (29.3%). All patients have provided written consent for the use of their medical data. The study was granted approval by our Institutional Review Board and was conducted according to the declaration of Helsinki.

Clinicopathological, treatment, and survival data were collected from patients’ records. More specifically, demographical data including patients’ date of birth, age at diagnosis, and date of first disease progression and/or death were collected. Type of surgery included primary or interval debulking, and surgery outcome, where available, was defined as optimal or suboptimal. Data regarding chemotherapy regimens and ICI therapy or combination were also collected. PFS and OS were calculated as part of the survival analysis. These were calculated as the number of months from the date of cancer diagnosis until disease progression or the date of death, respectively.

Evaluation of endocrine and other irAEs was performed according to CTCAE v4.03. Baseline evaluation for thyroid dysfunction is performed in all patients starting immunotherapy in our center and is periodically monitored during their treatment. Therefore, thyroid dysfunction could be detected as part of routine evaluation. Hypophysitis and other endocrine complications are suspected on the basis of relevant clinical symptoms, when additional testing is performed as appropriate (6).

### Statistical Analysis

All data were coded and analyzed by a specifically designed database of the SPSS statistical package (SPSS Inc., Armonk, NY) version 24. The Kolmogorov–Smirnov test was used to assess the regularity of the data. Overall survival (OS) was defined as the time between the time of diagnosis and the date of death from any cause. Progression-free survival (PFS) was defined as the time between the time of diagnosis and the date of progression. Alive patients were censored at the date of last contact.

Kaplan–Meier estimates were used to describe and visualize the effect of categorical variables on OS and PFS. Survival analysis was performed by Kaplan–Meier curves, and survival differences between groups were estimated using the log-rank test. The estimation of the prognostic value of several variables with patients’ survival was made by Cox regression models. Multivariate Cox regression analysis was used to estimate the independent predictive value of the various factors in patients’ survival. All statistical correlations were considered significant in case of p < 0.05.

## Results

### Patients’ Characteristics

Characteristics of the patients are presented in **Table 1**. In total, 351 patients were included in the analysis, 248 men (70.7%) and 103 women (29.3%). The median age was 66 years. Patients had a variety of cancer types, namely, bladder cancer (131, 37.3%), renal cancer (89, 25.4%), lung cancer (74, 21.1%), ovarian cancer (22, 6.3%), and others (35, 10%). In the other types of category, pancreatic cancer (3 patients), breast cancer (2 patients), uterine cancer (7 patients), colon cancer (4 cases), hepatocellular cancer (1 patient), cervical cancer (6 patients), prostate cancer (3 patients), melanoma (2 patients), and multiple myeloma (7 patients) were included. The majority (314, 89.4%) were classified as stage IV, while 10.6% (37) were classified as stage III. Most of the patients received immunotherapy with anti-PD1 agents (262, 74.6%) and the rest with anti-PD-L1 agents (89, 25.4%).

**Table 1:** Patients’ characteristics.

N	351
**Age** (median, 25th–75th percentile)	66.0, 58.0–72.0
**Gender**	
Males	248 (70.7%)
Females	103 (29.3%)
**Cancer type**	
Bladder cancer	131 (37.3%)
Renal cancer	89 (25.4%)
Lung cancer	74 (21.1%)
Ovarian cancer	22 (6.3%)
Other	35 (10.0%)
**Stage**	
III	37 (10.6%)
IV	314 (89.4%)
**Type of immunotherapy**	
Anti-PD1	262 (74.6%)
Anti-PD-L1	89 (25.4%)
**Patients with endocrine complications**	68 (19.4%)
Thyroid dysfunction	66 (18.8%)
Hypophysitis	1 (0.3%)
Thyroid dysfunction and hypophysitis	1 (0.3%)
**ECOG-PS**	
0–1	285 (81.2%)
≥2	55 (15.7%)
Missing	11 (3.1%)

In total, 68 (19.4%) of patients presented an endocrine complication after immunotherapy with ICIs. Specifically, 66 (18.8%) had thyroid dysfunction, 1 patient presented hypophysitis (0.3%), and 1 patient had a combination of thyroid dysfunction and hypophysitis (0.3%). Of note, endocrine irAEs were more common in renal carcinoma (25/89, 28.1%), urothelial cancer (28/131, 21.3%), and ovarian cancer (5/22, 22.7%) patients and less common in lung cancer (7/74, 9.5%) and other neoplasm (4/35, 11.4%) (p = 0.029) patients.

The median time of onset of endocrine complications was 10 weeks (range 14 days to 7.5 months). Most cases were treated with an appropriate hormonal supplementation. In the case with both hypophysitis and thyroid dysfunction, ICI immunotherapy was suspended. In two cases of thyroiditis, both levothyroxine and short-term corticosteroid therapy was used.

### Survival

**Figure 1:**
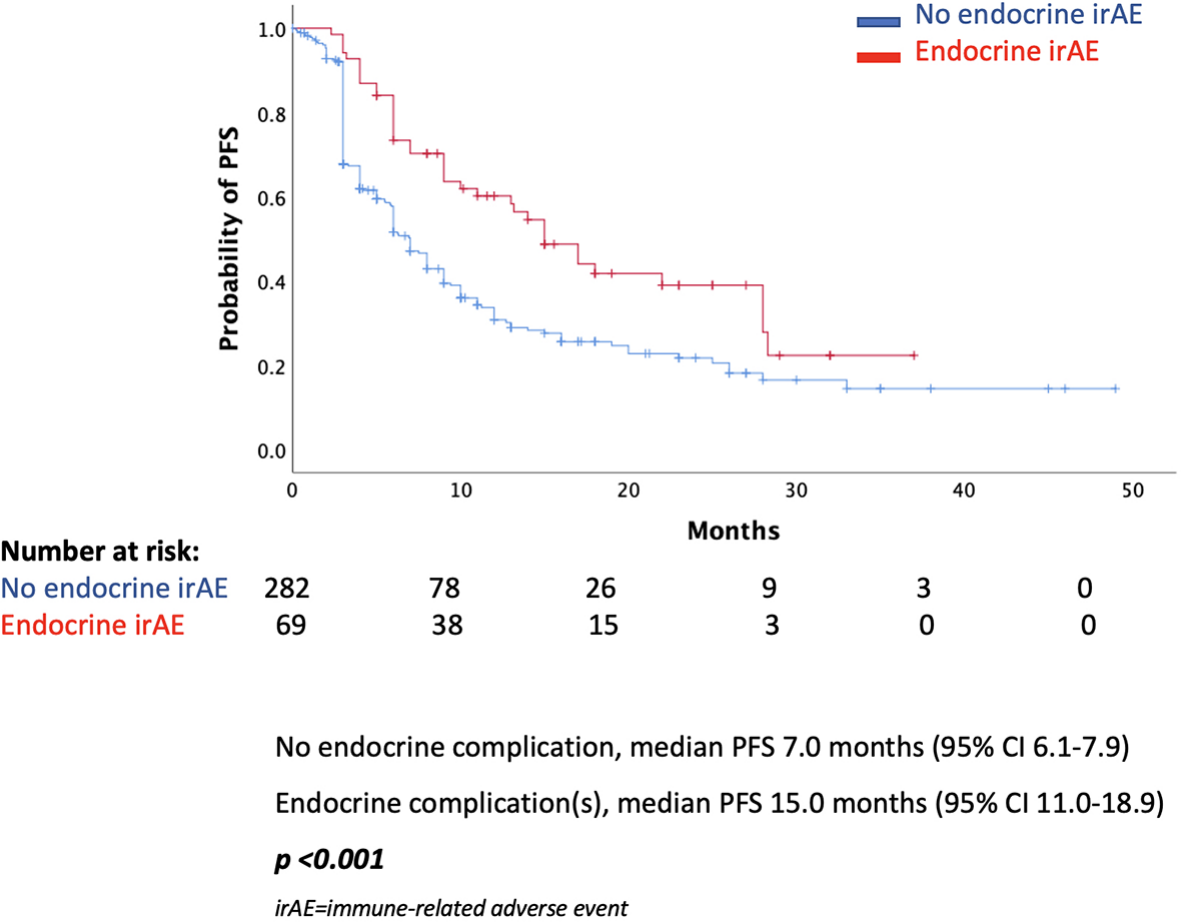
Progression-free survival according to the presence of endocrine complications.

**Figure 2:**
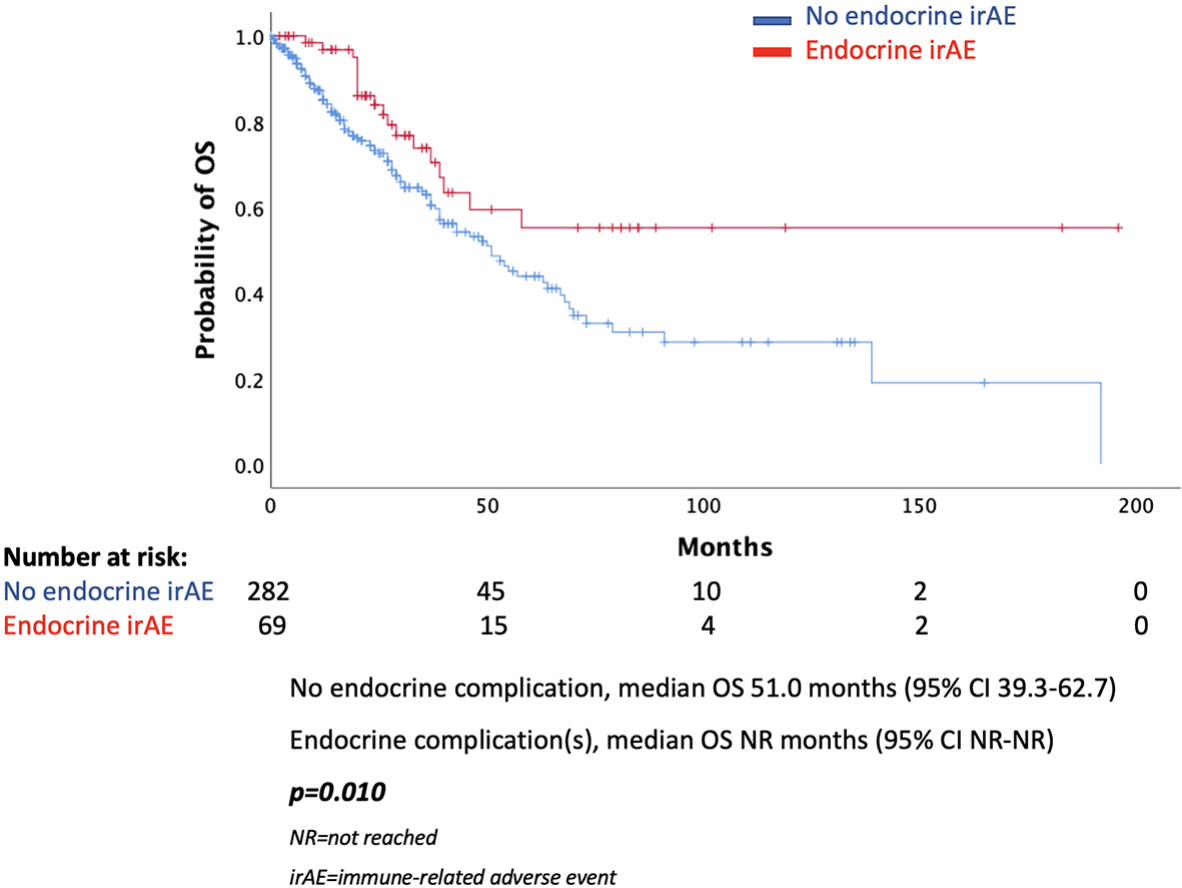
Overall survival according to the presence of endocrine complications.

In the total population, there was a statistically significant difference in median PFS (mPFS) between those presented an endocrine complication and those who did not. More specifically, patients with an endocrine irAE had mPFS of 15 months (95% CI 11.0–18.9 months), while in those without endocrine complication mPFS was 7 months (95% CI 6.1–7.9 months, *p* < 0.001) (**Figure 1**). Similarly, median OS (mOS) was statistically significantly lower in the patients’ group without endocrine complication. In fact, mOS was 51 months (95% CI 39.3–62.7 months) for these patients, while mOS was not reached for patients with endocrine complications (**Figure 2**).

**Figure 3:**
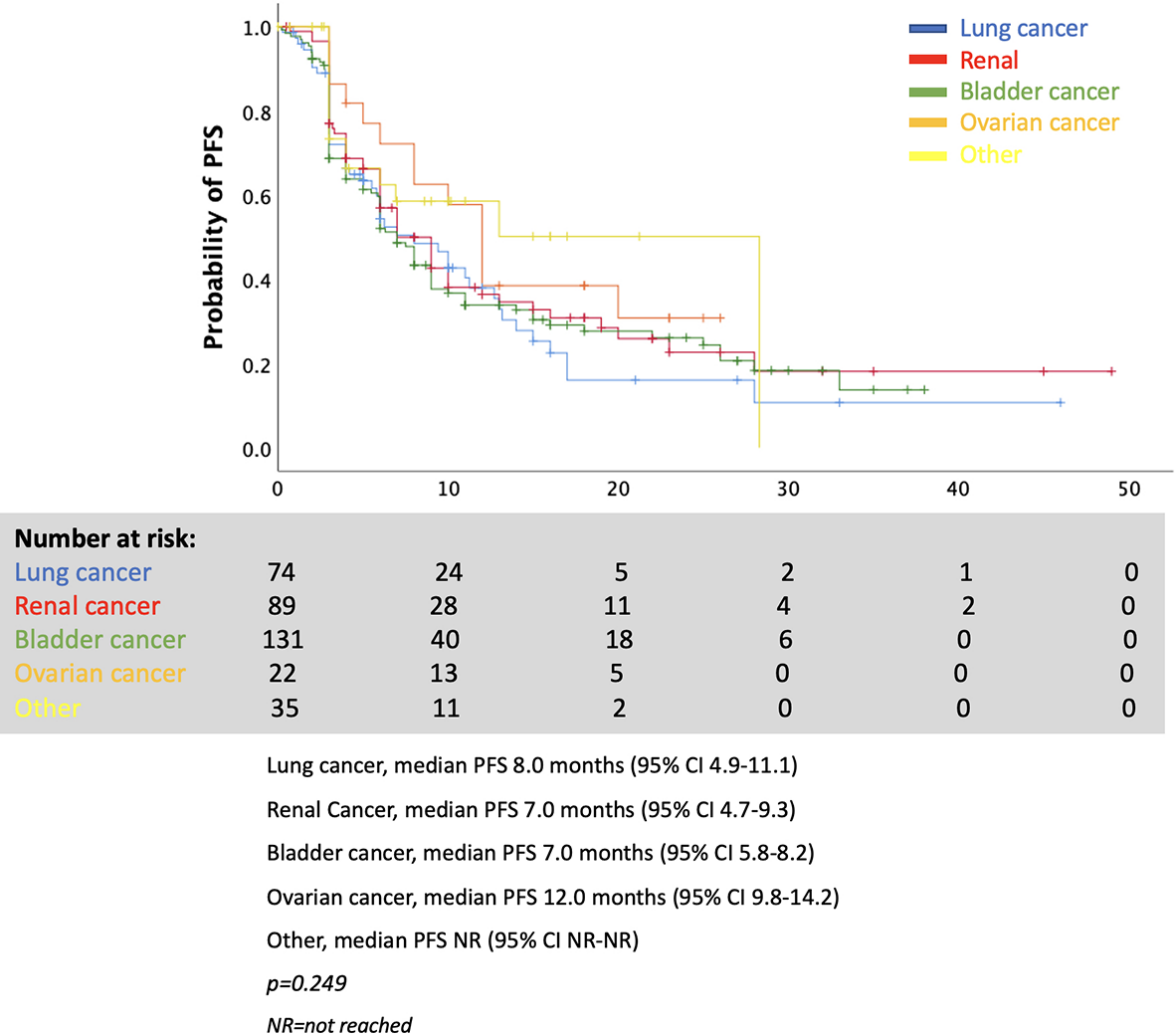
Progression-free survival by cancer type.

**Figure 4:**
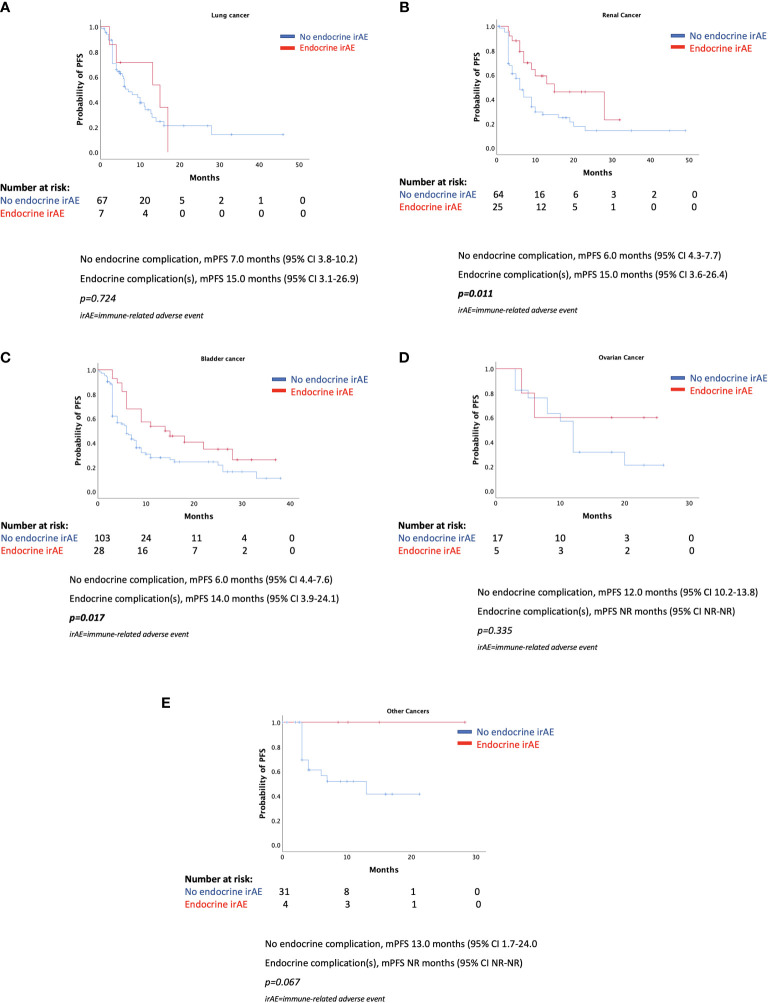
**(A–E)** Progression-free survival according to the presence of endocrine complications by cancer type.

As shown in **Figure 3**, the various mPFSs in patients of different cancer types are presented. There are no statistically significant differences. When examining the effect of endocrine irAE presence of mPFS and mOS in patients with various cancers separately, we found that patients with renal cancer and bladder cancer who presented endocrine complications after ICIs had longer PFS. However, results were not statistically significant for patients with lung cancer, ovarian cancer, or other types of cancer (**Figures 4A–E**).

In order to further elucidate the findings in the whole group, we performed multivariate analysis for PFS (**Table 2**) and OS (**Table 3**), including a number of parameters, such as gender, stage, type of immunotherapy, type of cancer, ECOG performance status scale, and endocrine complication. Interestingly, the presence of endocrine complications after immunotherapy with ICIs retained its significance in terms of longer PFS (HR 0.57, 95% CI 0.39–0.81) (**Table 2**) and OS (HR 0.53, 95% CI 0.32–0.90) (**Table 3**).

**Table 2:** Multivariate analysis for progression-free survival (PFS).

Variable	HR (95% CI)	*p value*
***Gender***		0.078
Male	1	
Female	0.71 (0.49–1.04)	
***Stage***		0.008
III	1	
IV	2.02 (1.19–3.40)	
***Type of immunotherapy***		0.042
Anti-PD1	0.70 (0.49–0.98)	
Anti-PD-L1	1	
***Type of cancer***		0.077
Lung cancer	1	
Renal cancer	1.02 (0.67–1.52)	
Bladder cancer	1.16 (0.76–1.74)	
Ovarian cancer	0.49 (0.25–0.99)	
Other	0.57 (0.30–1.05)	
***Endocrine complication***		0.002
No	1	
Yes	0.57 (0.39-0.81)	
***ECOG-PS***		0.709
0–1	1	
≥2	0.93 (0.63–1.37)	

**Table 3:** Multivariate analysis for overall survival (OS).

Variable	HR (95% CI)	*p value*
***Gender***		0.527
Male	1	
Female	0.86 (0.53–1.39)	
***Stage***		0.151
III	1	
IV	1.70 (0.70–3.53)	
***Type of immunotherapy***		0.016
Anti-PD1	0.56 (0.35–0.89)	
Anti-PD-L1	1	
***Type of cancer***		0.002
Lung cancer	1	
Renal cancer	0.36 (0.19–0.65)	
Bladder cancer	0.77 (0.43–1.37)	
Ovarian cancer	0.45 (0.16–1.24)	
Other	0.29 (0.12–0.69)	
***Endocrine complication***		0.018
No	1	
Yes	0.53 (0.32–0.90)	
***ECOG-PS***		0.877
0–1	1	
≥2	0.96 (0.55–1.66)	

## Discussion

The aim of this study was to investigate the association of endocrine complications after ICI immunotherapy with PFS and OS in a large cohort of oncological patients. We observed that 19.4% of participants presented endocrinopathies in total. Oncological patients with an endocrine complication after ICIs had longer mPFS compared with those without endocrine complication. Similarly, median OS was statistically significantly lower in the patients’ group without endocrine complication. These results retained significance even after multivariate analysis, including gender, stage of cancer, type of immunotherapy, and type of cancer.

The results of our study are in accordance with other recent findings, proposing that autoimmune complications after ICIs may be a positive predictor of therapy response. A recent large meta-analysis (13) included 30 (15–44) studies with 4,971 individuals and provided evidence that patients with autoimmune complications after ICIs experienced both an OS benefit and a PFS benefit compared with patients who did not develop such complications [OS: hazard ratio (HR) 0.54, 95% CI 0.45–0.65; p < 0.001; PFS: HR 0.52, 95% CI 0.44–0.61, p < 0.001). Specific analysis regarding endocrine complications yielded similar results (OS: HR 0.52, 95% CI, 0.44–0.62, p < 0.001). The same was found for dermatological (OS: HR 0.45, 95% CI 0.35–0.59, p < 0.001) and low-grade irAEs (OS: HR 0.57, 95% CI 0.43–0.75; p < 0.001) (13). Another later multicenter cohort study confirmed that the development of multisystem immune-related adverse events was associated with improved survival in patients with advanced (stage III/IV) NSCLC treated with anti-PD-L1 agents (12).

We need to note that a definite conclusion on the topic has not been drawn yet, as several single studies have provided negative or contradictory results (34–46). However, recent and increasing evidence, including our findings, suggests that such complications can be a positive predictor of immunotherapy response, despite the undesirable symptoms. The exact mechanisms of the association between autoimmune complications and survival benefits are not completely clear. Such adverse events are thought to represent bystander effects from activated T lymphocytes. An immune response against both the tumor and healthy cells or tissues seems to take place, probably through antigen mimicry procedures. The humoral immunity dysregulation has been additively proposed as a possible explanation for the phenomenon. For example, the PD-1 signaling pathway modulates B cell activation in both T-cell-dependent and -independent pathways (10, 13).

There is a term in the literature known as “beneficial autoimmunity.” Importantly, the nature of “beneficial autoimmunity” is substantially influenced by the type of cancer treated. For example, the induction of vitiligo in the case of melanoma is a good prognostic sign, while the repigmentation is associated with progression. In contrast, the presentation of pneumonitis in a patient with lung cancer would indicate poor prognosis. The difference reflects the fact that autoimmune responses that lead to the eradication of non-essential cells, such as melanocytes, are affordable to the body whereas damage of essential tissues, such as respiratory cells in the lung, is not (10). This is in accordance with the data from the recent meta-analysis that confirmed the association of endocrine, dermatological, and low-grade autoimmune complications with favorable outcomes (13). Of note, thyroid dysfunction is considered the most frequent ICI complication, irrespective of the location of the primary tumor (2, 6, 10, 13). Similarly, in our study most patients presented with thyroid dysfunction. Specifically, 66 (18.8%) had thyroid dysfunction, 1 patient presented hypophysitis (0.3%), and 1 patient had a combination of thyroid dysfunction and hypophysitis (0.3%).

In our study, most of the patients received immunotherapy with anti-PD1 agents (262, 74.6%) and the rest with anti-PD-L1 agents (89, 25.4%). Interestingly, the recent meta-analysis (13) showed that the association between autoimmune complication development and a favorable benefit on survival was significant in those patients undergoing treatment with anti-PD1 agents (OS: HR 0.51, 95% CI, 0.42–0.62, p < 0.001), but not with CTLA-4 inhibitors (OS: HR 0.89, 95% CI, 0.49–1.61, p = 0.706). In the meta-analysis, 26 studies adopted anti-PD-1 agents, 3 anti-CTLA-4 agents, and 1 anti-PD-1/PD-L1 agents (13). We need to take into consideration the lower numbers regarding CTLA-4 inhibitors; however, there may be a pathophysiological explanation too. Indeed, CTLA-4 inhibition activates T cells at an earlier stage of their development. Therefore, such an inhibition may directly disrupt central tolerance without affecting tumor immune response at the same time (1, 6, 13).

We found no statistically significant differences in mPFS of different cancer types, while the presence of endocrine complications was a positive predictor for longer mPFS in patients with renal cancer and bladder cancer, but not in patients with lung cancer, ovarian cancer, or other types of cancer. We need to note that numbers of the latter patients’ subgroups may be low to prove such an outcome for each different type of cancer. Indeed, several previous studies have shown favorable outcomes for patients with melanoma and lung cancer who developed various irAEs after immunotherapy with ICIs (12, 13, 15–17, 19–23, 25, 26).

The main strength of the study is the large number of participants. This is one of the largest cohorts in the literature. All patients come from the same academic unit, which implies another strength—that all participants were followed up by the same multidisciplinary team and with the same protocol. Numbers may be low for separate types of cancers, and this can be considered as a limitation. Moreover, data are retrospectively analyzed, however deriving from a prospectively collected cohort. Last but not least, a potential survivorship bias may exist for the development of irAEs.

In conclusion, this study provided evidence that oncological patients who present endocrine complications after ICI immunotherapy had longer mPFS and mOS compared with those without endocrine complication. These results retained significance even after multivariate analysis, including gender, stage of cancer, type of immunotherapy, and type of cancer. Therefore, physicians dealing with such patients should be aware that endocrine complications after ICIs may be a positive predictor of immunotherapy response and a prognostic factor of longer PFS and OS.

## Data Availability Statement

The raw data supporting the conclusions of this article will be made available by the authors, without undue reservation.

## Ethics Statement

The studies involving human participants were reviewed and approved by the Alexandra Hospital Ethics Committee. The patients/participants provided their written informed consent to participate in this study.

## Author Contribution

SP designed the protocol and wrote the manuscript. ML participated in the collection of the data and performed the statistical analysis. EE-P participated in the collection of the data. KS participated in the collection of the data. CM participated in the collection of the data. KK participated in the collection of the data. FZ participated in the collection of the data and revised the manuscript. TP participated in the collection of the data and revised the manuscript. M-AD designed the protocol and revised the manuscript. All authors contributed to the article and approved the submitted version.

## Conflict of Interest

The authors declare that the research was conducted in the absence of any commercial or financial relationships that could be construed as a potential conflict of interest.

## Publisher’s Note

All claims expressed in this article are solely those of the authors and do not necessarily represent those of their affiliated organizations, or those of the publisher, the editors and the reviewers. Any product that may be evaluated in this article, or claim that may be made by its manufacturer, is not guaranteed or endorsed by the publisher.
